# Engineered Common Cold Virus Helps Cultured Cystic Fibrosis Tissues Clear Mucus

**DOI:** 10.1371/journal.pbio.1000160

**Published:** 2009-07-21

**Authors:** Robin Meadows

**Affiliations:** Freelance Science Writer, Fairfield, California, United States of America

**Figure pbio-1000160-g001:**
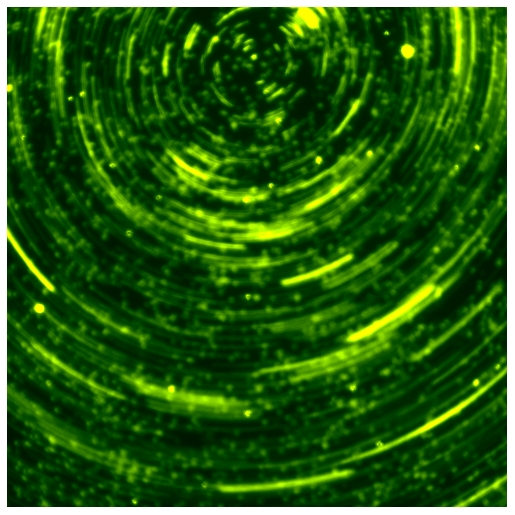
Normal mucus transport is restored in ciliated airway cultures derived from patients with cystic fibrosis after replacement of the *CFTR* gene. This image represents yellow fluorescent beads embedded in mucus spinning on top of a culture with bead trails, visualized by long exposure times. (Beads moved at 40 µm/sec, approximating mucus velocity in vivo.) (Image: Raymond J. Pickles).

[Fig pbio-1000160-g001]The lung disease cystic fibrosis (CF) has the dubious distinction of being the most common fatal recessive hereditary illness among people of Northern European descent. Most patients die of respiratory failure because their lungs are not adequately protected from airborne bacteria and other pathogens. In healthy people, cells lining the airways ward off infection by making thin, slippery mucus that traps pathogens and is moved out of the airways via cilia, so it can be coughed out of the lungs. In contrast, CF patients have thick, sticky mucus that builds up in the airways, resulting in repeated infections, lung damage, and respiratory failure. The disease is caused by mutations in an ion channel gene called the CF transmembrane conductance regulator (*CFTR*). These mutations disrupt the ion balance of the airway lining fluid, leading to dehydrated mucus. The defective *CFTR* also affects other mucus-secreting glands, notably in the pancreas, as well as sweat glands.

Current lung treatments for CF patients include chest pounding and aerobic exercise to loosen the mucus, and medicines to thin the mucus and limit infection. While there have been efforts to treat the underlying cause of the disease with gene therapy, so far they have not delivered enough *CFTR* to the airway lining (or epithelial) cells to help CF patients in clinical trials.

In this issue of *PLoS Biology*, Raymond Pickles and colleagues report a new gene therapy approach that increases the delivery of *CFTR* to human ciliated airway epithelial (HAE) cells that are collected from patients and grown in the laboratory. This culture model simulates the airway lining in people and has predicted the outcomes of other gene therapy approaches. The surfaces of cultured HAE cells from healthy people both produce and transport mucus, while those from CF patients retain both the ion channel dysfunction and dehydrated mucus that are characteristic of the disease. To introduce *CFTR* into HAE cells, the researchers inserted the gene into human parainfluenza virus (PIV), which causes common colds and other respiratory infections. The researchers had shown in 2005 that PIV targets ciliated HAE cells, which compose about 70% of the airway surface lining and normally express *CFTR*, making them good candidates for delivering *CFTR* to CF patients.

In the current study, PIV with the *CFTR* insert (PIVCFTR) infected about 60% of the ciliated CF cells within five minutes, suggesting that short exposures might be able to treat CF patients effectively. To confirm that PIVCFTR corrected the ion channel dysfunction in these cells, the researchers stimulated them with the CFTR activator forskolin. Bioelectric measurements revealed that the ion channel activity was restored to normal levels, which came as a surprise because mRNA and protein analyses had shown that the treated cells produced at least 100 times more CFTR protein than normal, leading the researchers to expect an exaggerated jump in ion channel activity. A possible explanation is that regardless of CFTR protein levels, cell membranes can contain only so many of these ion channels.

PIVCFTR restored airway surface liquid levels in cultures from CF patients to those of cultures from healthy people. Untreated ciliated CF cells absorbed so much liquid that its depth was reduced to 3 µm in 48 hours, while treated cells maintained liquid depths of about 8 µm, which is in the normal range. Moreover, PIVCFTR increased the beat frequency of CF cilia: untreated cilia beat only about twice per second, while treated cilia beat at the normal eight beats per second. These observations suggest that rehydrating the mucus is enough to restore effective beating of the ciliated CF cells that line airways. Most compellingly, PIVCFTR also increased mucus transport in ciliated CF cells, which the researchers measured by dropping 1-µm fluorescent beads onto the cultures. The beads did not move at all on untreated CF cells, while the beads formed bands indicating that the mucus moved at about 20 µm per second on treated cells. However, this is only about half the normal rate of mucus transport, which the researchers suggest may be due to subtle cytotoxic effects of PIV replication.

Finally, the researchers asked how many airway lining cells must receive CFTR to restore mucus hydration and transport in cultured CF airway cells. Infecting about a quarter of the cells was enough to restore normal liquid depths, and infecting about 40% was enough to restore normal mucus transport. The next step of determining how many cells must be infected in CF patients would require testing PIVCFTR on living organisms. The problem is that PIV is a human pathogen but most animal models of CF are in mice, which are not infected by PIV. Because a newly developed pig model may not be susceptible to the virus either, the best test of PIVCFTR may be on CF patients.

Another drawback to using PIVCFTR is that infected ciliated cells are shed from the airway lining within a week, presumably as a natural defense against the virus. The researchers are hopeful that PIV can be engineered so it no longer triggers, or at least delays, this cell shedding, perhaps by deleting viral envelope glycoproteins that may be cytotoxic. But because the normal lifespan of these ciliated cells is just 90–120 days, PIV-based CF treatments would have to be ongoing in any case. Despite the hurdles that must be overcome before PIVCFTR could be used on people, this study is a welcome advance in the quest for an effective treatment of this disease that cuts lives short.

**Zhang L, Button B, Gabriel SE, Burkett S, Yan Y, et al. (2009) CFTR Delivery to 25% of Surface Epithelial Cells Restores Normal Rates of Mucus Transport to Human Cystic Fibrosis Airway Epithelium. doi:10.1371/journal.pbio.1000155**

